# Supplementary figures and images for: Identification and classification of Aquilaria (Thymelaeaceae): inferences from a phylogenetic study based on matK sequences
Source: PeerJ. 2025 Jul 23;13:e19752. doi: 10.7717/peerj.19752 (PMC12296565; doi:10.7717/peerj.19752)

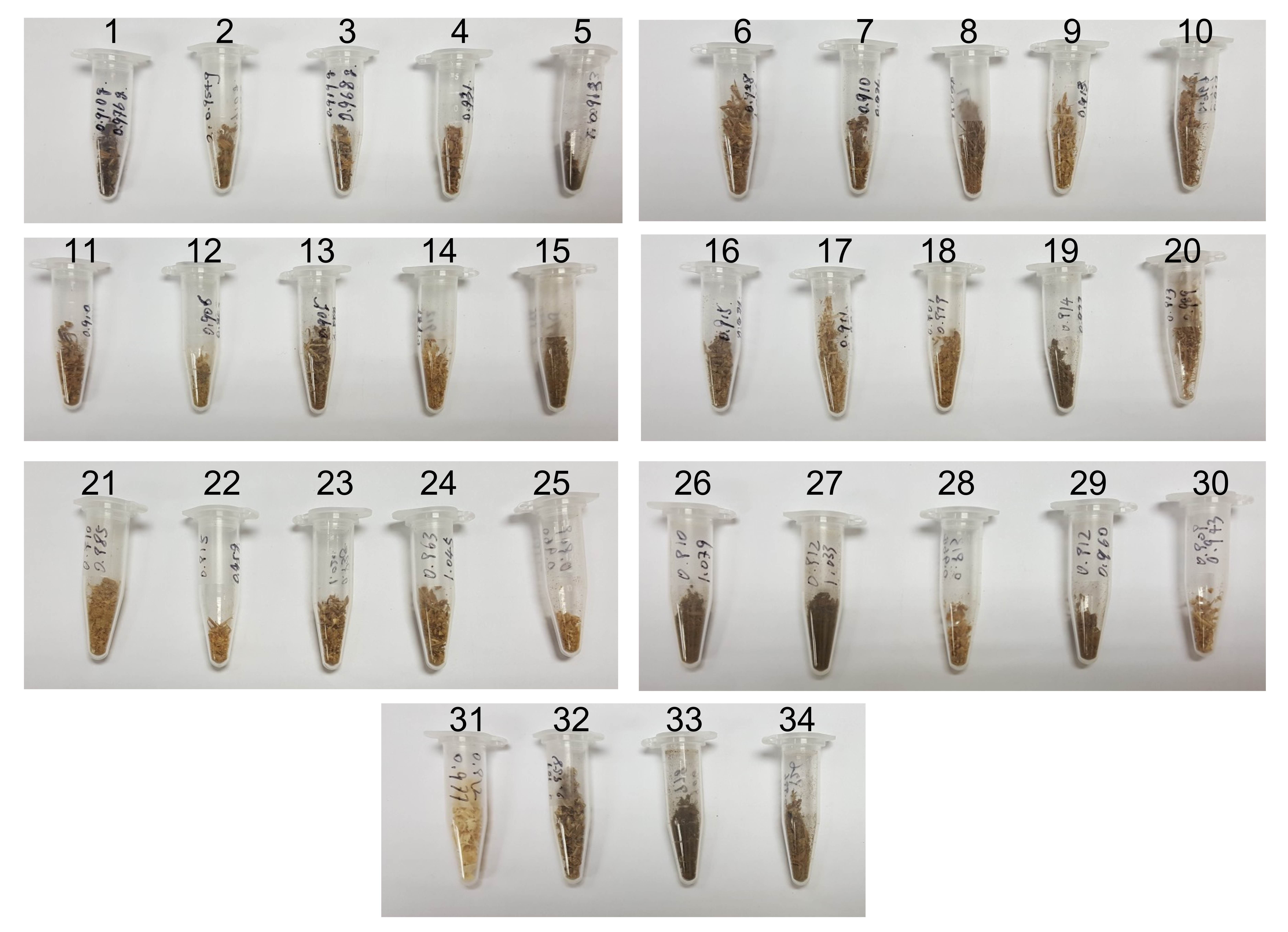

Supplement: Supplemental Information 1 [file peerj-13-19752-s001.png]

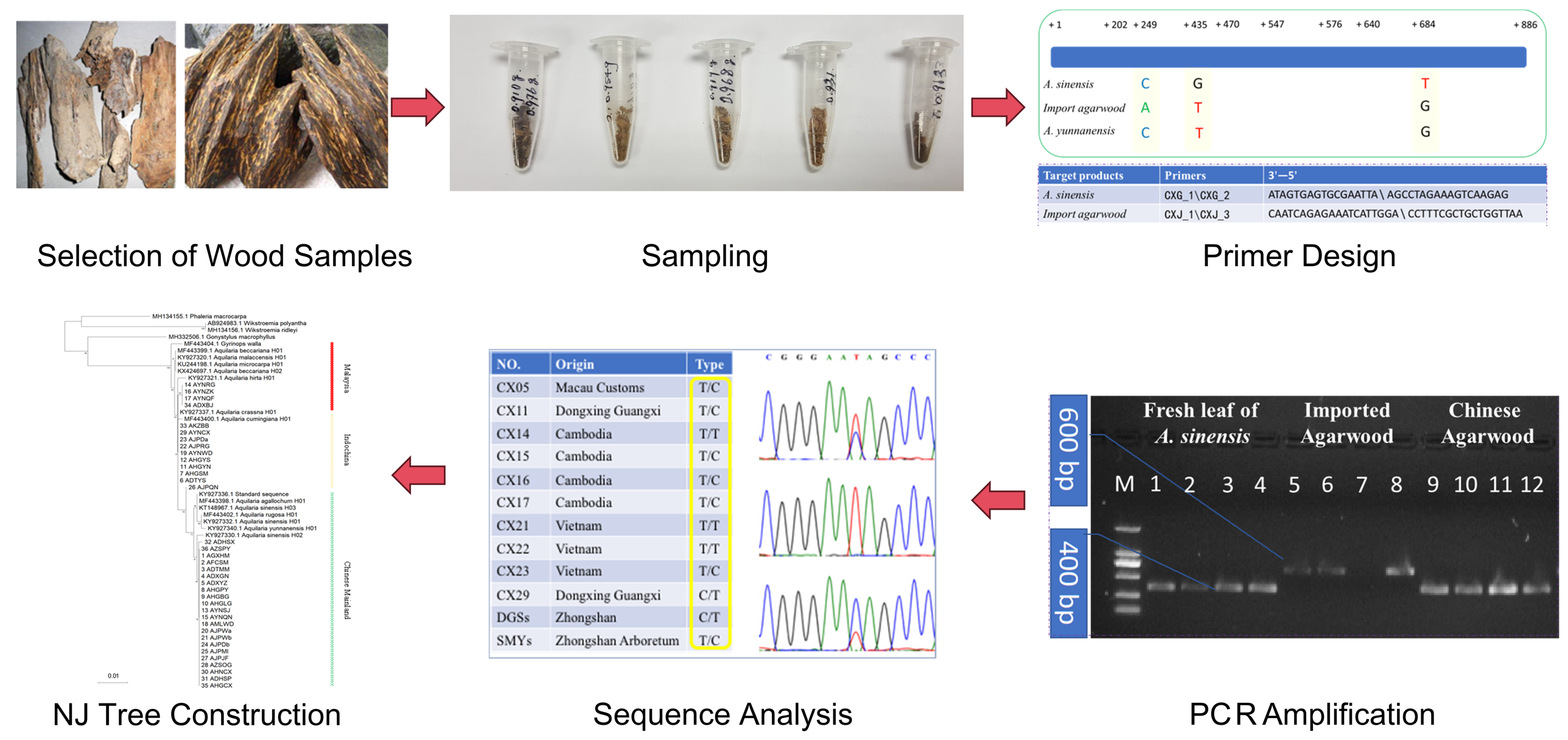

Supplement: Supplemental Information 2 [file peerj-13-19752-s002.png]

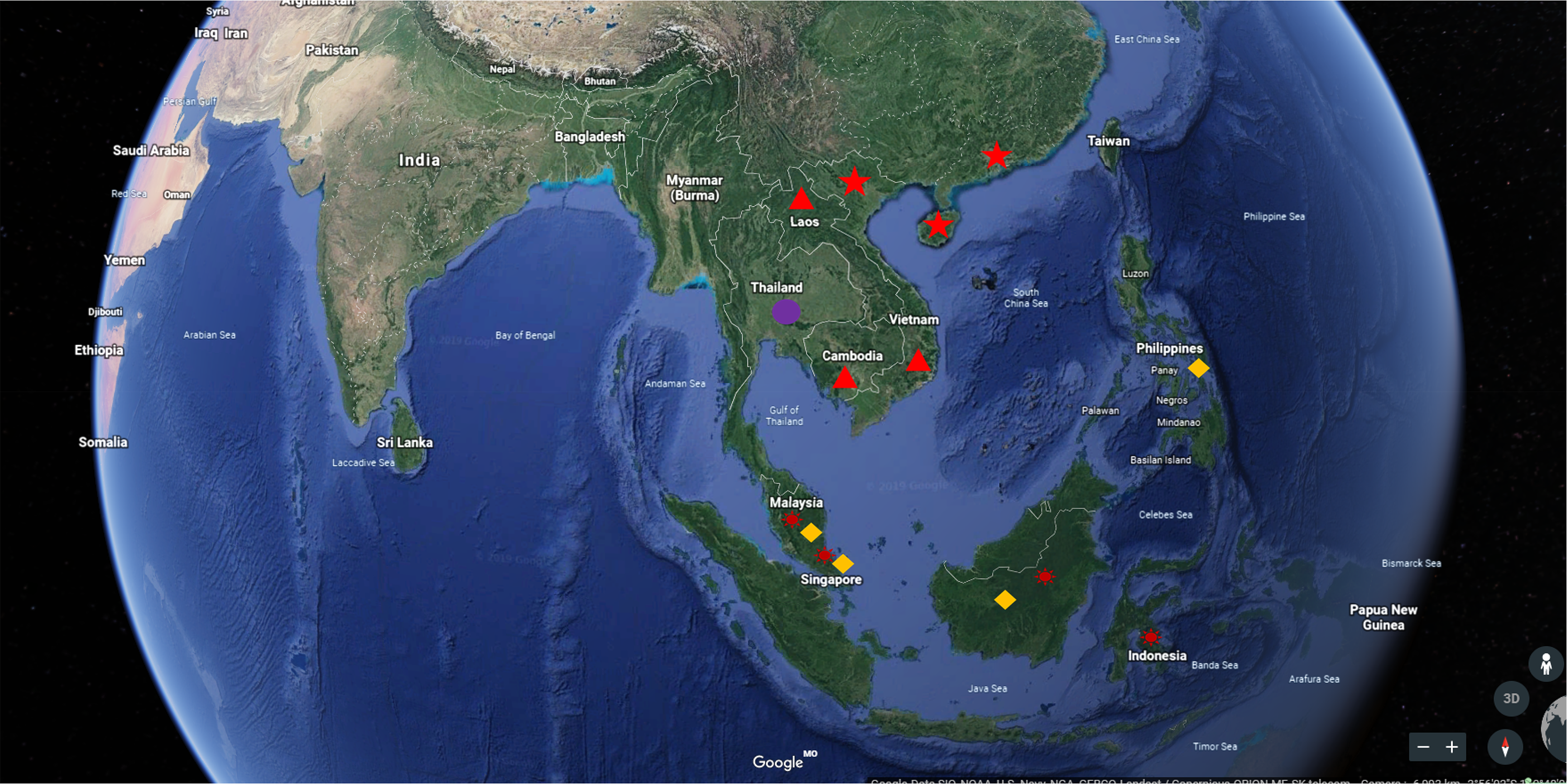

Supplement: Supplemental Information 3 — The triangle denotes A. crassna, the circle represents A. subintermedia, the prism symbolizes A. malaccensis, the sun-shaped symbol indicates A. hirta, and the pentagram signifies A. sinensis. [file peerj-13-19752-s003.png]

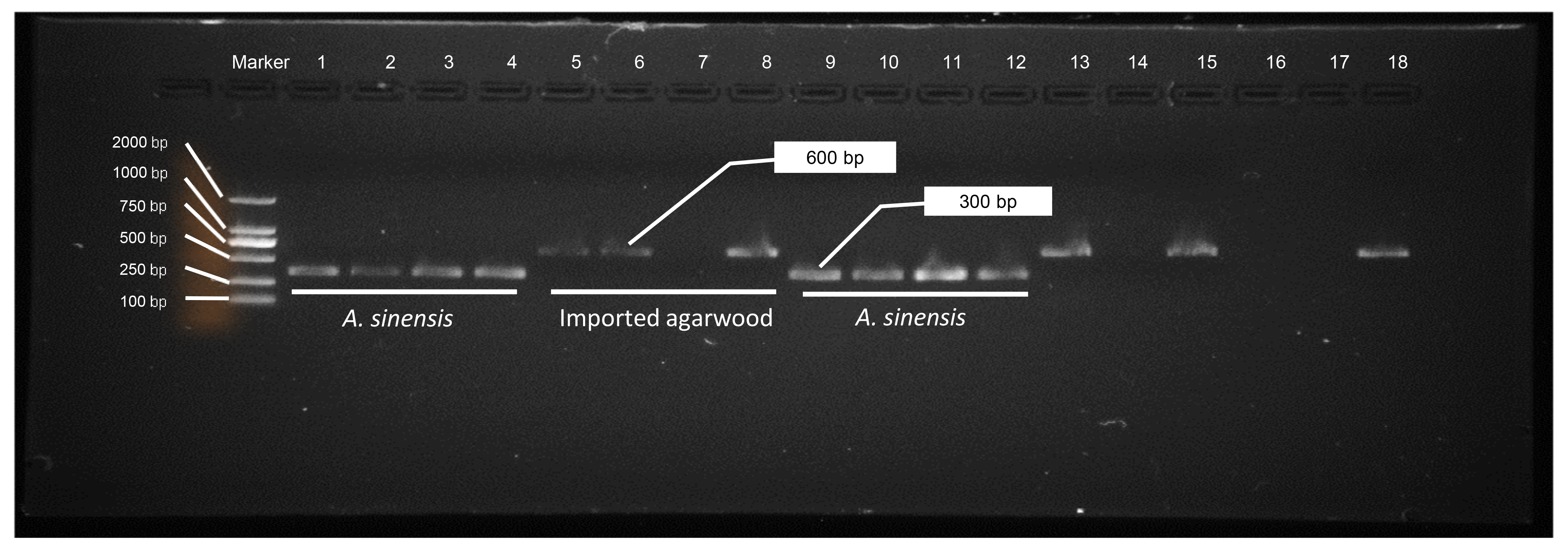

Supplement: Supplemental Information 4 [file peerj-13-19752-s004.png]

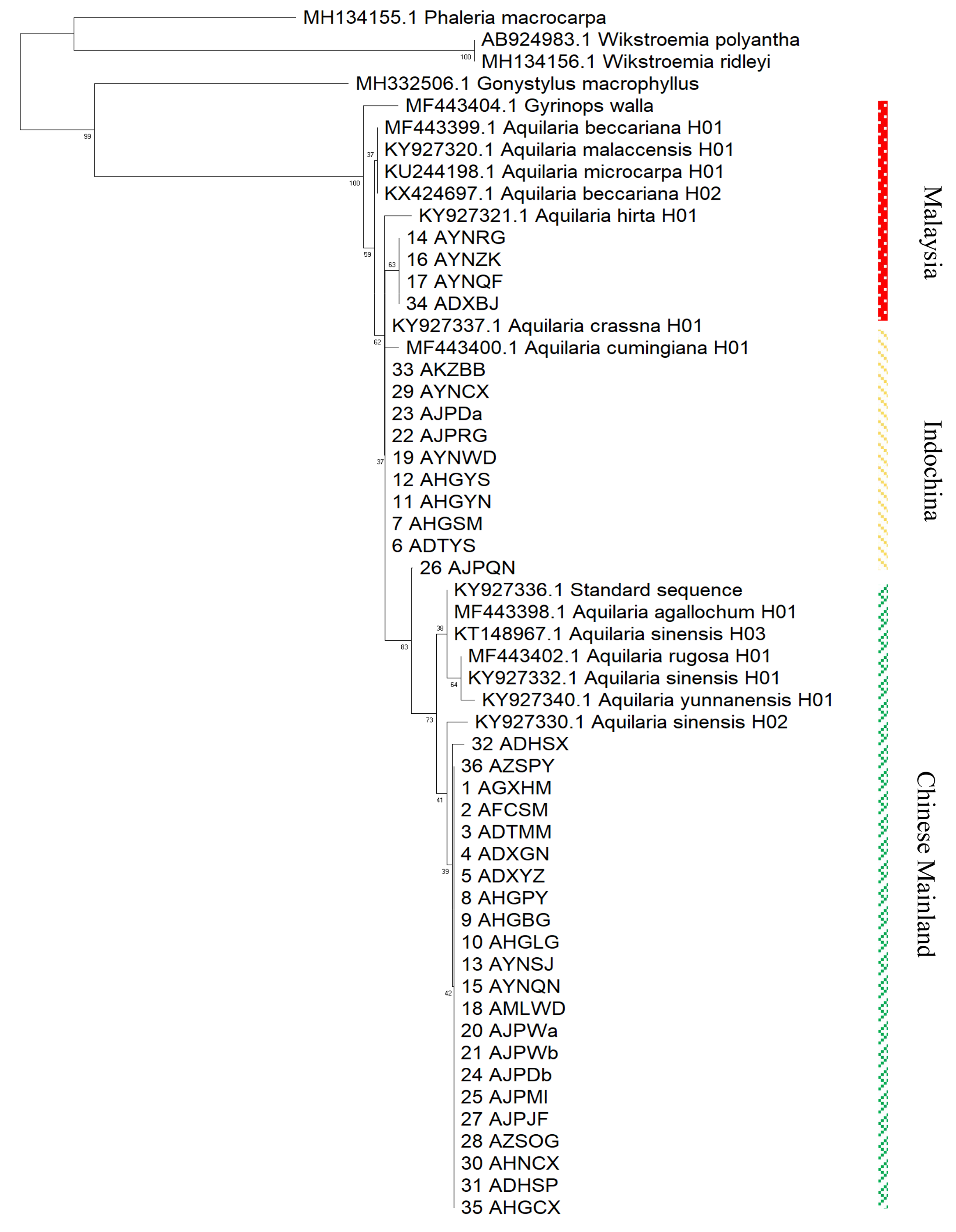

Supplement: Supplemental Information 5 [file peerj-13-19752-s005.png]

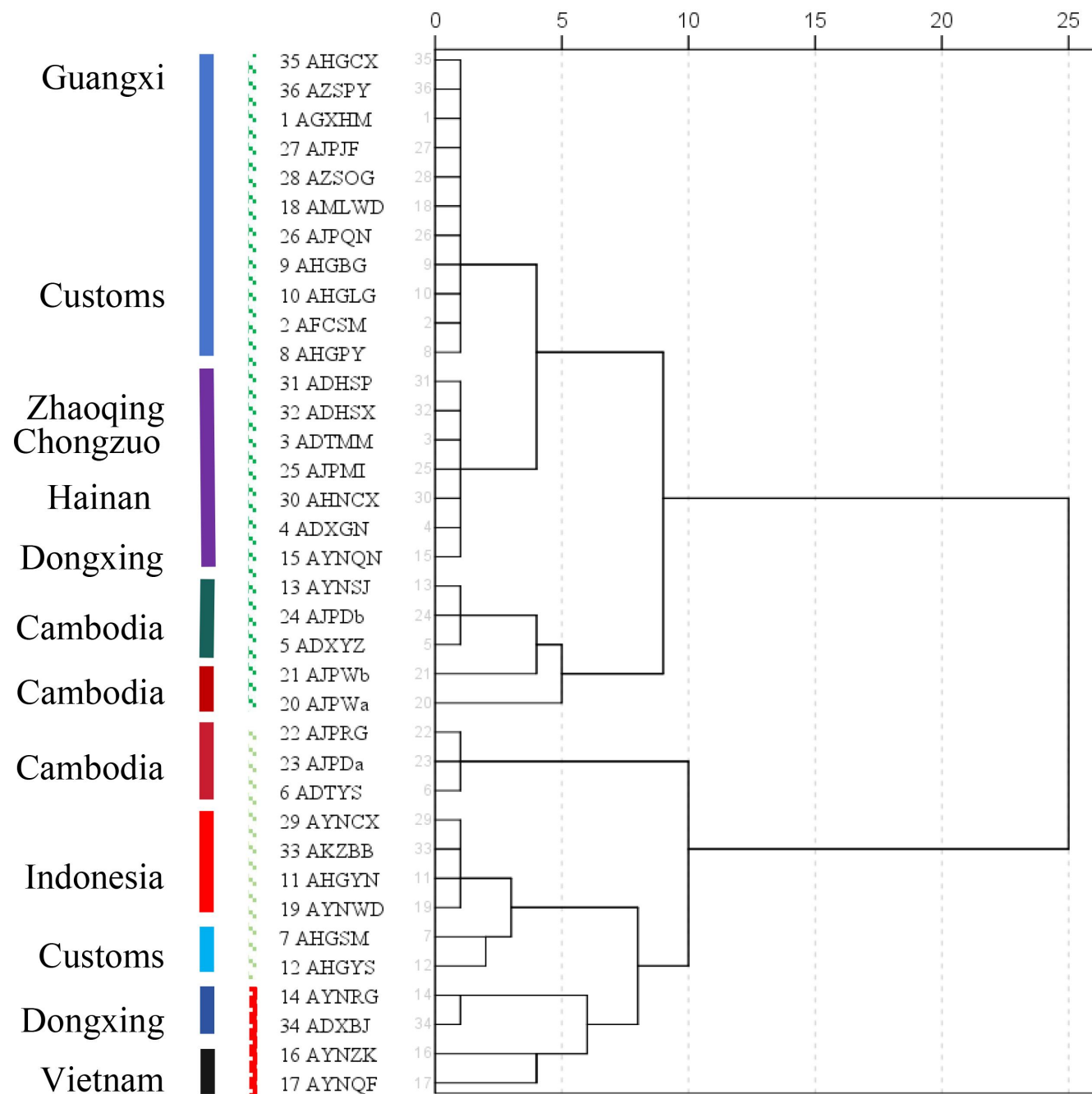

Supplement: Supplemental Information 6 [file peerj-13-19752-s006.pdf]

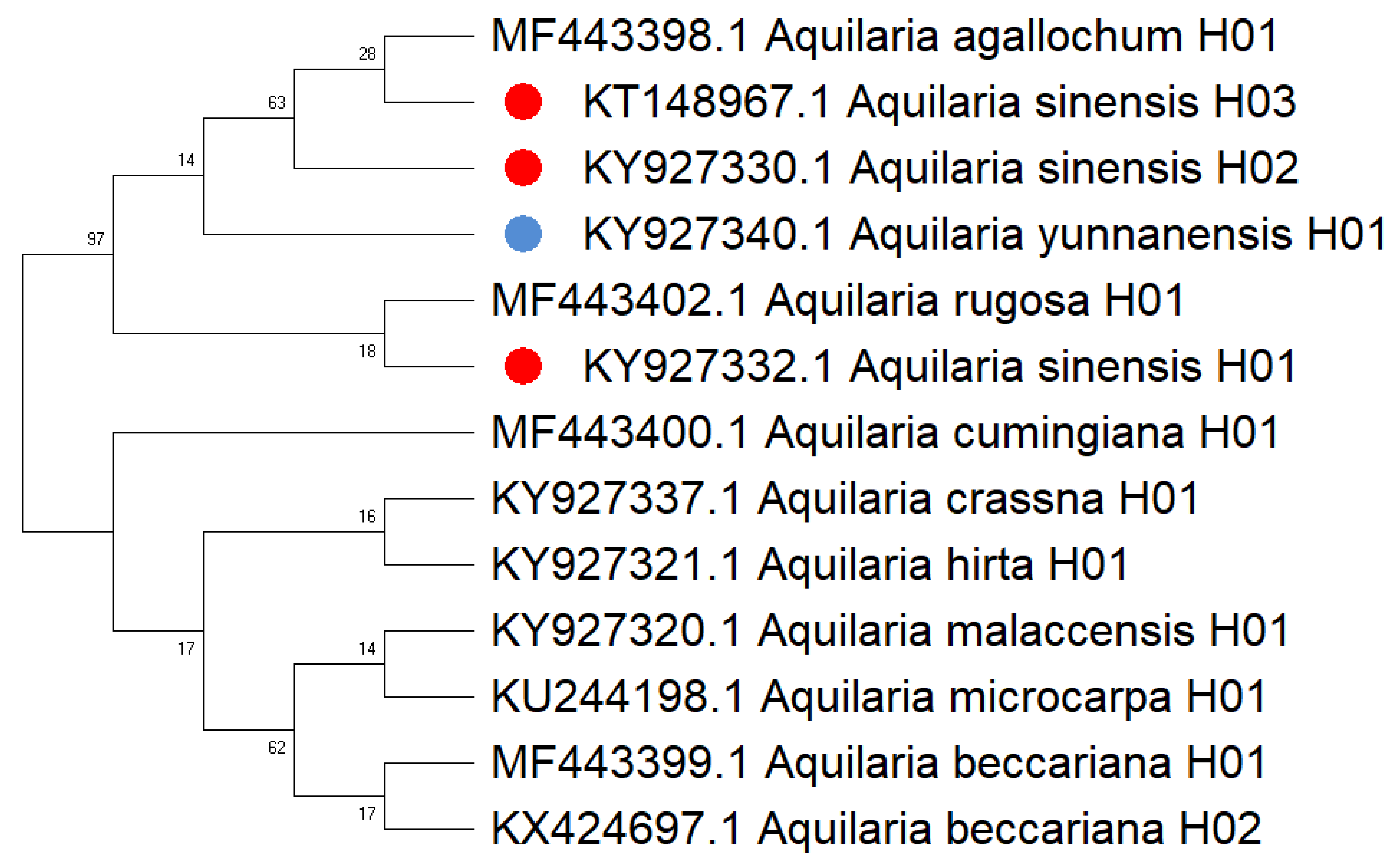

Supplement: Supplemental Information 7 [file peerj-13-19752-s007.png]
